# Linguistic evidence supports date for Homeric epics

**DOI:** 10.1002/bies.201200165

**Published:** 2013-02-18

**Authors:** Eric Lewin Altschuler, Andreea S Calude, Andrew Meade, Mark Pagel

**Affiliations:** 1) Departments of Physical Medicine and Rehabilitation, and Microbiology and Molecular Medicine, University of Medicine and Dentistry of New Jersey, University HospitalNewark, NJ, USA; 2) School of Biological Sciences, Lyle Building, University of ReadingReading, UK; 3) Santa Fe InstituteSanta Fe, NM, USA

**Keywords:** dating, evolution, Homeric epics, Iliad, linguistics, phylogeny, statistics

## Abstract

The Homeric epics are among the greatest masterpieces of literature, but when they were produced is not known with certainty. Here we apply evolutionary-linguistic phylogenetic statistical methods to differences in Homeric, Modern Greek and ancient Hittite vocabulary items to estimate a date of approximately 710–760 BCE for these great works. Our analysis compared a common set of vocabulary items among the three pairs of languages, recording for each item whether the words in the two languages were *cognate* – derived from a shared ancestral word – or not. We then used a likelihood-based Markov chain Monte Carlo procedure to estimate the most probable times in years separating these languages given the percentage of words they shared, combined with knowledge of the rates at which different words change. Our date for the epics is in close agreement with historians' and classicists' beliefs derived from historical and archaeological sources.

The Homeric epics are among the greatest masterpieces of literature. The Iliad's story of the Trojan Wars tells us that the epics were almost certainly produced sometime after the 12th century BCE – if indeed the wars were ever fought – but the question is how much later? Herodotus thought considerably later: Writing in the Histories Book II.53 around 450 BCE, he stated that Homer ‘lived, as I believe, not more than 400 years ago’. The most commonly accepted date among modern classicists, drawing on historical, literary and archaeological analyses, is around the mid-8th century BCE 1, 2, although some authors propose a more recent 7th century BCE date 3.

Here, we investigate whether formal statistical modelling of languages can help to inform this historical question. In particular, we investigate whether evolutionary-linguistic statistical methods can be usefully applied to differences in Homeric, Modern Greek and ancient Hittite vocabulary items to provide a date for these great works.

## Cognate words and rates of lexical change

Languages, like biological species, commonly evolve by a process of ‘descent with modification’ 4. The most obvious way that languages change is to change their words or vocabulary. For instance, the English *dog* is a new word that has largely replaced the Old English *hund* to specify that meaning: the modern English *hound* is now reserved for particular breeds (e.g. Irish Wolfhound, Scottish Deerhound), dogs used for hunting, and idiomatic references to dog(s), for example ‘her faithful hound’, rather than to dogs in general.

In the study of such lexical change, the basic unit of analysis is the cognate. Cognates are words that derive from a common ancestral word, just as in biology homologous genes derive from a common ancestral gene. For example, cognates meaning ‘water’ exist in English (*water*), German (*wasser*), Swedish (*vatten*) and Gothic (*wato*), reflecting descent from proto-Germanic (*watōr).

The Old English *hund* is cognate to *hound*, but the newer word *dog* represents an instance of what is known as *lexical replacement*. In previous work 5, we have produced statistical estimates of rates of lexical replacement for a range of vocabulary items in the Indo-European languages. A word's rate of lexical replacement measures the instantaneous rate at which a word such as *hund* gets replaced over time by a new unrelated or non-cognate word such as *dog*.

We derived our estimates of lexical replacement from a formal statistical model applied to a widely studied set of common vocabulary items known as the Swadesh 200-word list 6. The rates were evaluated by studying how the words for each meaning in the Swadesh list evolved along the branches of a phylogenetic tree of 87 Indo-European languages 5.

We found that words reliably differ in their rates of lexical replacement. Most words have a 50% chance of being replaced by a new non-cognate word every 2,000–3,000 years (a word's linguistic *half-life* 5), but among the words in the Swadesh list there is at least a 100-fold variation in rate of change, such that some have linguistic half-lives under a millennium whereas others are expected to last over ten thousand years.

The variation in rates of lexical replacements makes these vocabulary items promising candidates for estimating the time of divergence between pairs of languages: rapidly evolving words might be expected to change even among relatively closely related languages, whereas more slowly evolving words might remain cognate even among distantly related languages – if all or none of the words changed, establishing a date would be impossible. More generally, given a set of rates and a set of vocabulary items that have either remained cognate or not between pairs of languages, we can ask what divergence times best satisfy the observed distribution of cognacy relations among those languages.

**Figure 1:**
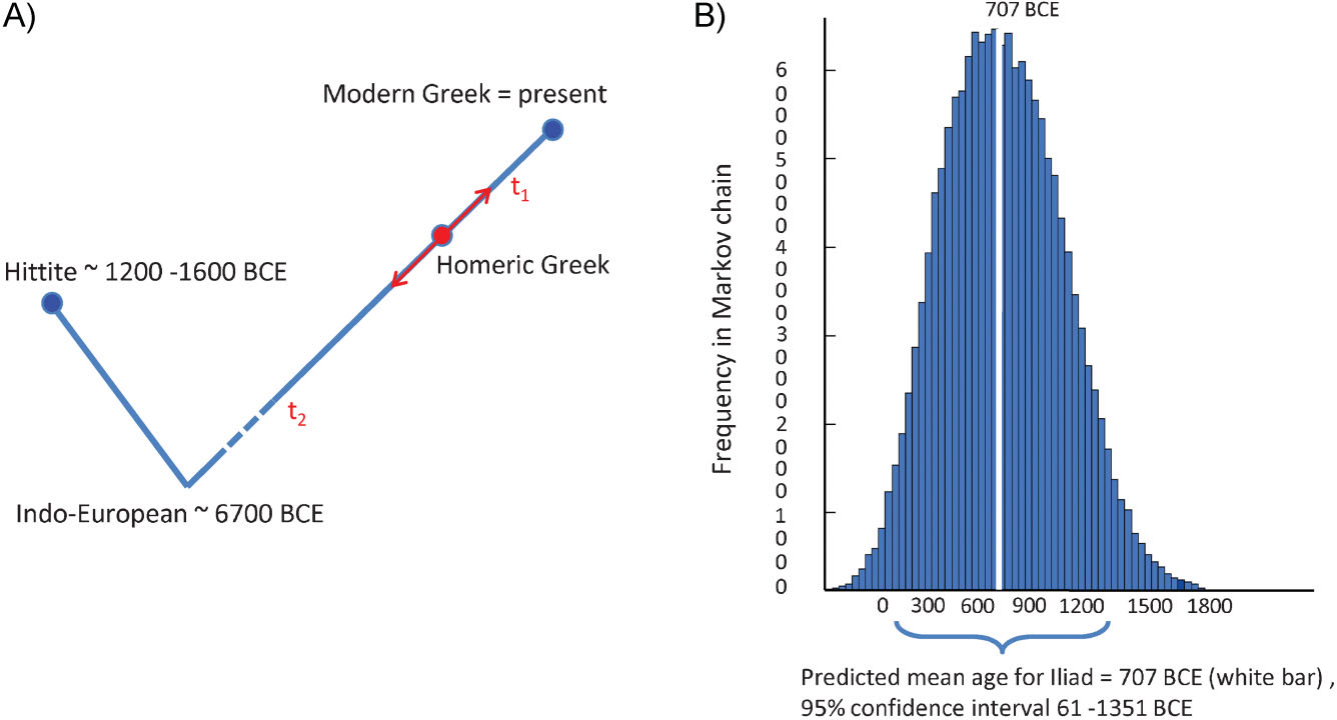
**A:** Evolutionary (phylogenetic) relationships among Hittite, Homeric Greek and Modern Greek with approximate ages shown (note: lines not to scale). Indo-European refers to the root or origin of this family of languages. **B:** frequency histogram of estimated ages for Homer (t_1_) showing posterior mean estimated age for the Homeric epics (white bar). Age for Indo-European from 12.

We apply that logic here to estimate the age of the Homeric epics, using comparisons among three sets of vocabulary items – Hittite, and Homeric and Modern Greek. Hittite is an extinct language in the Anatolian branch of Indo-European. Preserved cuneiform scripts dating to the 13th to 16th centuries BCE provide a source of its vocabulary. The Anatolian branch of Indo-European is an outgroup to the main Indo-European languages containing Homeric and the later Modern Greek (Fig. 1A), and so Hittite is expected to be widely divergent from the other two. Together, then, comparisons among Hittite, and Homeric and Modern Greek ‘bracket’ Homeric Greek, providing overlapping evidence to where in time Homer's works fall between them (branch denoted ‘*t*_1_’ in Fig. 1A).

We compiled data on cognacy relationships for the items in the Swadesh 200-word list 7–11, separately for the three language pairs. We associated with each word its rate of lexical replacement (the rate at which a word is replaced by a new unrelated or non-cognate word) in the Indo-European languages (taken from 5).

We then estimated the time separating pairs of vocabulary sets by seeking the times in years that simultaneously maximized the likelihood of observing these distributions of cognacy judgements, on the phylogeny in Fig. 1A, given their rates of lexical replacement. Given *n* = 173 words, the likelihood (*L*) that any two languages *i* and *j* are separated by *t* units of time given the cognacy data *D* is:


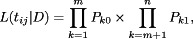


where 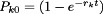, 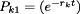, *m* corresponds to words in the Swadesh list that we scored as not cognate between any two language families, *n*–*m* counts the words scored as cognate, and *r*_*k*_ is the rate of change for the *k*^th^ word as estimated in the Indo-European languages. The overall likelihood is the product of the three pairwise comparisons.

## An inferred date for the Homeric epics

We were able to extract information about shared cognates among the three language-pairs for 173 of the 200 Swadesh items. Consistent with expectations, Hittite and Modern Greek share the fewest cognates (13.3%), followed by Hittite and Homeric Greek at 19.1% of their vocabulary, and the figure for Homeric and Modern Greek is 50% (Table 1). In each case, the words remaining cognate between a pair have slower rates of lexical replacement than those that have changed (Table 1, all *p*-values <0.0001). The mean difference in rates of replacement is greatest for the youngest pair (Modern-Homer) where change is restricted largely to rapidly evolving words, and least for the oldest pair (Hittite-Modern) where even many slowly evolving words have been replaced owing to the greater length of time they have been separated.

**Table 1:** Comparisons among Hittite, and Homeric and Modern Greek

Data	Hittite-Homer	Hittite-Modern Greek	Homer-Modern Greek
Cognate/not cognate^a^	33/140	23/150	87/86
Percent cognate	19.1	13.3	50.3
Replacement rate for cognate^b^	1.62 ± 1.26 (sd)	1.28 ± 1.03	1.19 ± 1.63
Replacement rate for non-Cognate	3.32 ± 1.77	3.26 ± 1.76	3.54 ± 1.83

a Refers to number of words out of 173 (see text) judged cognate between the two languages;

b Rates of lexical replacement taken from 5.

We derived a posterior distribution of dates for Homer from a Bayesian Markov chain Monte Carlo method (Fig. 1B) that proposed times for *t*_1_ while simultaneously integrating over two prior distributions of dates, one for the Indo-European root and the other for Hittite. The posterior distribution returns a posterior mean estimate of the date for Homer's works of 707 BCE, with 95% confidence intervals (sometimes denoted credible intervals) ranging from 61 BCE to 1351 BCE. The upper confidence interval does not rule out a far earlier date for the epics than is commonly believed, but suggests (Fig. 1B) that it is unlikely they were produced near to the time of the Trojan Wars. The lower (younger) limit of approximately 61 BCE might seem absurd given historical evidence and beliefs, but is not wholly implausible on linguistic grounds alone, even if – as Fig. 1B shows – it is improbable.

## Conclusions

Our analysis gives a formal quantitative estimate of a date for the Homeric epics that agrees with the commonly accepted 8th century BCE origin of these great works. Our posterior distribution of dates is also consistent with Herodotus' remark that would place the epics around 850 BCE, but would treat as unlikely any suggestion that Homer might have been a ‘war correspondent’ recording the events of the Trojan War as they happened, if indeed they ever did (of course, the epics were originally an oral tradition so we cannot know if our dates reflect when they were produced or when they were eventually written down).

Our analysis is not informed or constrained in any way by historical, cultural or archaeological information about Homer or his works, being derived solely from information on shared cognates among Hittite, and Homeric and Modern Greek, and rates of lexical replacement in Indo-European languages. In spite of this, our estimated date falls roughly in the middle of the classicists' and historians' preferred date for Homer, representing a prediction spanning nearly three millennia. This, along with the consistency of the results (Table 1), demonstrates a remarkable regularity in the ways that words are replaced over time, and illustrates that language can be used, like genes, to aid in the investigation of questions in history, archaeology and anthropology 13.

Our Bayesian approach is easily extended to include other sources of information, such as might be obtained from historical accounts. For instance, given that Herodotus was aware of Homer, we might have restricted our search (Fig. 1B) to times earlier than 450 BCE. As an illustration, we have repeated our Bayesian analysis using a normally distributed prior for the age of Homer centred on 800 BCE with a standard deviation of 200 years. This makes a 450 BCE or even younger date unlikely but not impossible.

The new model returns a date for Homer of 762 BCE with 95% confidence intervals from 376 to 1157 BCE. It is intriguing in this light that the ‘Nestor's cup’ 14, a vase excavated from an Ancient Greek site in Italy, contains an inscription that some scholars think refers to a line from the Iliad, and is dated to ∼740–720 BCE. Equally, the new 95% upper limit falls in the middle of the 12th century, the period during which some scholars think the Trojan wars might have been fought.

Along with the historical and other accounts of Homer's great works, our analysis of common vocabulary items in the Iliad increases our confidence in its age, and shows how even fictional texts can preserve traces of history.
